# The Effectiveness of Topical Lactic Acid Gel in Episiotomy Wound Healing and Its Impact on Sexual Quality of Life After Childbirth: A Prospective Study

**DOI:** 10.7759/cureus.84517

**Published:** 2025-05-21

**Authors:** Dragos Brezeanu, Ana-Maria Brezeanu, Sergiu Chirila, Vlad I Tica

**Affiliations:** 1 6th Department, Faculty of Medicine, Ovidius University, Constanta, ROU

**Keywords:** episiotomy, lactic acid, perineal wound care, puerperium, quality of sexual life

## Abstract

Background: The postpartum period is essential for maternal recovery, significantly impacting both physical wound healing and the restoration of sexual function. Optimal episiotomy healing is directly correlated with improved postpartum sexual health, and lactic acid is being explored as a therapeutic agent in wound care due to its antimicrobial and regenerative properties. This study aims to evaluate the efficacy of topical lactic acid (Canesbalance®, Bayer, Leverkusen, Germany) in promoting episiotomy healing and its subsequent influence on postpartum sexual quality. Additionally, it explores how demographic factors, such as educational level, maternal age, and parity, affect postpartum sexual function.

Methods: We conducted a prospective comparative study involving 100 postpartum women divided into two groups: an experimental group treated with topical lactic acid gel applied to the episiotomy site seven days postpartum and a control group undergoing spontaneous wound healing without intervention. Episiotomy healing quality and sexual function were assessed once at 40 days postpartum using a 12-item sexual quality of life questionnaire. This was the only follow-up point in the study, and no additional clinical visits or evaluations were conducted beyond the 40-day postpartum assessment. Statistical analyses included independent t-tests to compare healing scores, ANOVA for evaluating demographic influences on sexual function, and correlation analyses between healing quality and postpartum sexual function scores.

Results: The lactic acid-treated group demonstrated significantly improved episiotomy healing scores compared to the control group. Educational level significantly influenced postpartum sexual quality, with higher education correlating with improved sexual function. Maternal age and parity did not demonstrate statistically significant effects on postpartum sexual function.

Conclusion: Topical lactic acid application significantly improves episiotomy wound healing and positively impacts postpartum sexual function. Educational attainment emerged as an important determinant of sexual quality postpartum. These findings advocate for further studies to confirm the long-term benefits and wider clinical applicability of lactic acid in postpartum care.

## Introduction

The postpartum period is a complex physiological and psychological transition during which optimal recovery plays a vital role in a woman’s health and quality of life [1]. Among obstetric interventions, episiotomy, a surgical incision made in the perineum during vaginal childbirth, is widely utilized to facilitate delivery and minimize severe perineal tears [2,3]. Nevertheless, inadequate or complicated healing of episiotomy wounds continues to represent a significant clinical issue, impacting maternal comfort, psychological well-being, and postpartum sexual health [4].

Episiotomy rates vary notably across Europe, with alarmingly high frequencies exceeding 60% in countries such as Cyprus, Portugal, Romania, and Poland, while markedly lower rates (below 10%) are reported in Denmark, Sweden, and Iceland [5]. Such substantial disparities underline an urgent need to reconsider obstetric practices to improve maternal outcomes and ensure safer childbirth experiences internationally [6].

Additionally, postpartum sexual dysfunction remains a common yet under-addressed issue, with contributing factors including educational background, maternal age, and parity [7]. Understanding the interplay between these demographic variables and wound healing quality can facilitate more targeted and effective postpartum interventions [8].

Although spontaneous healing is the conventional standard, there is increasing interest in therapeutic interventions capable of enhancing tissue regeneration and reducing postpartum discomfort. Lactic acid, recognized for its antimicrobial and regenerative properties, emerges as a promising candidate for accelerating perineal wound healing [9]. Previous research primarily focused on the role of lactic acid-producing bacteria and probiotics in tissue repair [10], but data specifically evaluating the direct effects of topical lactic acid on episiotomy recovery and postpartum sexual function remain limited.

The aim of this study was thus to address this knowledge gap by examining whether topical application of lactic acid can significantly improve episiotomy healing outcomes and positively impact postpartum sexual quality. We hypothesized that women receiving topical lactic acid treatment would demonstrate superior wound healing and enhanced postpartum sexual health compared to women undergoing spontaneous healing. The primary objective of this study was to evaluate the efficacy of topical lactic acid (Canesbalance®, Bayer, Leverkusen, Germany) in promoting episiotomy wound healing. The secondary objective was to assess the impact of this intervention on postpartum sexual quality of life. Additionally, we aimed to explore how demographic factors, such as educational level, maternal age, and parity, influence postpartum sexual function.

## Materials and methods

This prospective, non-randomized comparative controlled study was conducted from 01 February 2023 to 31 December 2024 at County Clinical Emergency Hospital “Sf. Ap. Andrei”, Constanța, Romania. Participants were sequentially allocated into two groups based on the order of inclusion and informed consent, without the use of randomization. Approval was obtained from the Ethics Committee of Ovidius University of Constanța (No. 01/20.01.2023) and Saint Andrew Hospital Ethics Committee (No. 7230/31.01.2023). Written informed consent was obtained from each participant before inclusion. Although the study was prospective, it was non-randomized and observational. Therefore, following national guidelines at the time, it was not registered in the Romanian Clinical Trials Registry. However, the study was approved by the Ethics Committees of both participating institutions and conducted in accordance with the Declaration of Helsinki.

**Figure 1:**
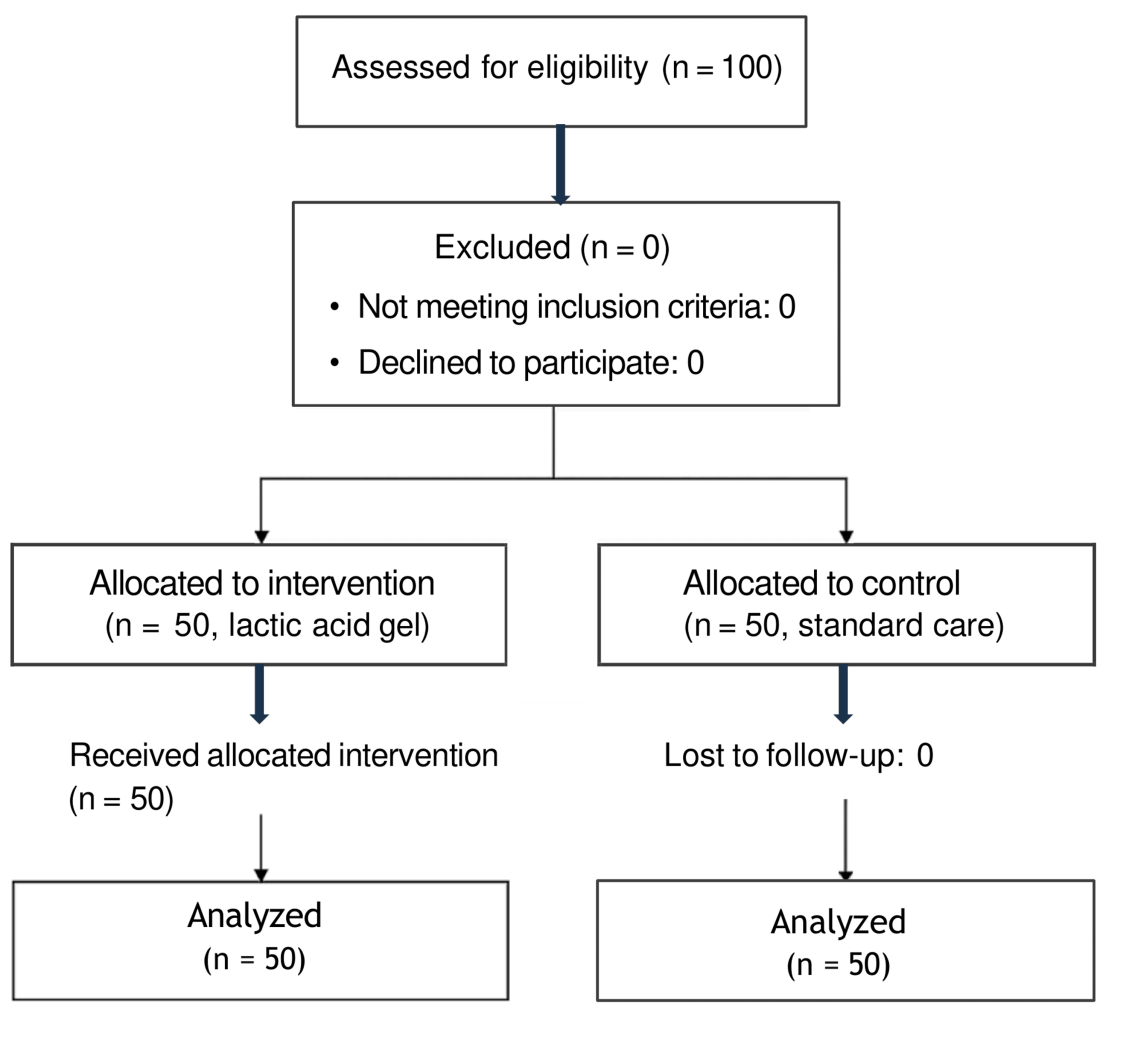
CONSORT Flow Diagram of Participant Recruitment and Allocation The figure illustrates the CONSORT flow diagram detailing the recruitment, screening, eligibility assessment, and sequential allocation of the 100 participants into the experimental and control groups. All eligible women who met the inclusion criteria and provided informed consent were enrolled consecutively until the target sample size was reached. No participants were lost to follow-up or excluded after allocation. CONSORT: Consolidated Standards of Reporting Trials

One hundred postpartum women aged 18-40 years, undergoing spontaneous vaginal delivery with mediolateral episiotomy at gestational ages of 38-40 weeks, were enrolled and sequentially assigned into two groups as shown in Figure 1.

The experimental group (n=50) received topical lactic acid gel (Canesbalance®, Bayer) applied once daily directly to the episiotomy site, beginning seven days postpartum, for seven consecutive days. The primary active ingredient used in the intervention was lactic acid. Canesbalance® (Bayer) also contains glycogen, sodium lactate, polyacrylate crosspolymer-6, hydrogenated palm glycerides citrate, and purified water. The gel has a pH of approximately 3.8, consistent with the natural vaginal environment. 

The control group (n=50) received standard postpartum perineal care as per institutional protocol, which included twice-daily external cleansing with warm water and mild soap, and the use of sterile perineal pads. No antiseptic or topical healing agents were applied. This standard care regimen was maintained for seven days postpartum, corresponding to the duration of treatment in the experimental group.

According to the institutional protocol, routine antibiotic therapy is not administered after uncomplicated vaginal deliveries with episiotomy. None of the participants in either the experimental or control group required or received antibiotics during the study period, as no signs of local or systemic infection were observed.

A formal a priori sample size calculation was not performed due to the exploratory nature of this study. Instead, a convenience sample of 100 women (50 per group) was used, based on recruitment feasibility within the study period and ethical approval limits. This approach aimed to identify preliminary efficacy signals to guide future research.

Inclusion and exclusion criteria

Eligible participants were postpartum women aged between 18 and 40 years who had undergone spontaneous vaginal delivery with a mediolateral episiotomy at term (gestational age 38-40 weeks). Only women without pre-existing wound healing disorders or infections at the time of delivery were considered for enrollment. Additionally, all participants were required to provide written informed consent before inclusion in the study.

Women were excluded if they had a known hypersensitivity to lactic acid-based products or if they had sustained severe perineal trauma, such as third- or fourth-degree tears. Further exclusion criteria included a history of gestational diabetes, immunosuppressive conditions, or the use of any additional wound care interventions beyond the standard hospital protocols.

Episiotomy healing quality and sexual function were assessed 40 days postpartum using a 12-item sexual quality of life questionnaire, scored on a 5-point Likert scale (Tables 6, 7 in the Appendices). Higher scores reflected better sexual health, with items 1-4 reverse-scored. All interventions were performed by standardized medical teams to ensure the uniformity of the procedure and the highest level of patient care. A dedicated statistician interpreted the data, ensuring a blind process and eliminating bias. Data were analyzed using IBM SPSS Statistics for Windows, Version 26 (Released 2019; IBM Corp., Armonk, New York, United States). Independent t-tests compared healing scores between groups, and analysis of variance (ANOVA) evaluated the effects of demographic variables. Correlation analysis examined relationships between wound healing scores and sexual function scores. Statistical significance was set at p<0.05, with Cohen’s d and η² calculated to assess effect size.

Applicability

The study focused on primiparous and second-parous patients, specifically those in the later stages of pregnancy, with gestational ages ranging from 38 to 40 weeks, as shown in Table 1. These participants underwent episiotomy procedures, and their wound was managed through a targeted medication regimen designed to promote healing and minimize complications. 

**Table 1:** Patient Demographics

Participants Group	Number of Participants (%)
Primiparius Control (%)	26 (26%)
Primiparous Treated (%)	32 (32%)
Secundi-parous Control (%)	24 (24%)
Secundi-parous Treated (%)	18 (18%)
Age 18-25 (%)	36 (36%)
Age 26-30 (%)	30 (30%)
Age 31-35 (%)	23 (23%)
Age 36-40 (%)	11(11%)
Low Education Level (%)	33 (33%)
Middle Education Level (%)	37 (37%)
High Education Level (%)	30 (30%)

## Results

The study included 100 postpartum women, divided evenly into two groups: 50 participants in the lactic acid group and 50 in the spontaneous healing (control) group. Sexual quality of life and wound healing were assessed 40 days postpartum using a validated 12-item questionnaire. 

A Pearson correlation analysis revealed a moderate positive correlation between episiotomy healing scores and postpartum sexual function scores across the entire cohort (r = 0.47, p < 0.001), indicating that better wound healing was associated with improved sexual function.

Comparison of overall sexual quality scores

Women treated with topical lactic acid gel reported significantly higher sexual quality scores compared to the control group, as shown in Table 2. The mean score for the lactic acid group was 41.66 ± 1.62, while the control group scored 40.74 ± 2.11. The difference was statistically significant (t = 2.46, p = 0.0156), indicating a beneficial effect of lactic acid treatment on postpartum sexual health.

**Table 2:** Comparison of Overall Sexual Quality Scores

Group	Mean ± SD	N
Lactic Acid Group	41.66 ± 1.62	50
Spontaneous Healing	40.74 ± 2.11	50

Influence of educational level on sexual function

ANOVA revealed that educational level significantly affected postpartum sexual quality (F = 3.77, p = 0.030), as highlighted in Table 3. Women with higher education levels reported better sexual health outcomes.

**Table 3:** Influence of Educational Level on Sexual Function

Education Level	Mean Score	Standard Deviation
Low	40.8	2.1
Middle	41.2	1.7
High	41.9	1.3

Effect of maternal age on sexual function

As seen in Table 4, there is no statistically significant difference found between age groups (F = 0.99, p = 0.379), suggesting that maternal age did not have a substantial impact on postpartum sexual quality in this cohort.

**Table 4:** Effect of Maternal Age on Sexual Function

Age Group (years)	Mean Score	Standard Deviation
18–25	41.3	2
26–30	41.5	1.8
31–35	41.7	1.7
36–40	41.2	1.9

Effect of parity on sexual function

Similarly to maternal age, as shown in Table 5, parity did not significantly influence sexual function scores (F = 1.24, p = 0.271). Both primiparous and secundiparous women reported comparable scores.

**Table 5:** Effect of Parity on Sexual Function

Parity	Mean Score	Standard Deviation
Primiparous	41.6	1.8
Secundiparous	41.4	1.9

## Discussion

The loss of libido is one of the most prevalent sexual dysfunctions encountered during the postpartum period, affecting numerous new mothers [11,12].

It's crucial to understand that each woman's journey is profoundly unique, and a diminished sex drive can have a significant impact on overall sexual satisfaction and intimacy within relationships [13,14].

Over 87% of pregnant women reported fatigue, a figure that rises to 95% during puerperium [15]. This fatigue, which can persist for up to one year after childbirth, is not just a physical burden but also significantly impacts the sexual health of new mothers [16]. 

The most frequently reported concerns immediately following delivery include pain and fatigue, as well as vaginal dryness and sexual desire disorders, which both affect 44% of women [17,18]. Our study aimed to assess the efficacy of lactic acid in promoting an increase in the quality of sexual life after puerperium. Lactic acid treatment did provide superior healing outcomes compared to natural recovery and increased the quality of sexual life in puerperium. The results indicate that lactic acid application significantly improves episiotomy healing compared to spontaneous healing. 

Additionally, many women report decreased self-esteem and anxiety about body changes during the early postpartum period, including discomfort from episiotomies, swelling, and perineal pain [19,20]. 

Vaginal dryness and dyspareunia have been shown to diminish quality of life and decrease the frequency of sexual activity [21,22]. Several studies confirm that perineal pain and sexual difficulties are significantly more common among primiparas [22,23]. These challenges notably affect perceived sexual satisfaction. In contrast, our study showed that age and number of births did not show significant associations with postpartum sexual function. This suggests that other factors, such as psychological well-being, partner support, and hormonal recovery, may have a stronger influence on sexual health.

During the postpartum period, the experience of orgasm can vary widely among women [24]. Some may find that their orgasms occur without substantial changes, while others may experience less intensity, or in some cases, may not orgasm at all. 

Factors influencing these variations can include hormonal fluctuations, physical recovery from childbirth, and emotional adjustments to motherhood [25]. Moreover, painful intercourse, known as dyspareunia, and the inability to achieve orgasm can lead to considerable distress, contributing to anxiety about resuming a fulfilling sexual relationship [26]. 

These concerns can create a cycle of fear and apprehension that further diminishes sexual desire. Studies found that women who faced difficulties during intercourse often experienced discomfort linked to conditions such as bladder and bowel incontinence, dyspareunia, and the presence of hemorrhoids [27]. These issues not only reduced their likelihood of engaging in sexual activity but also significantly impacted their overall quality of life [28,29].

Interestingly, education level was found to significantly impact postpartum sexual function. Women with higher education levels reported better sexual quality, potentially due to greater awareness of postpartum health, access to medical information, and proactive healthcare-seeking behavior [30]. Education level was not balanced across groups, with the lactic acid group having a higher proportion of participants with mid-to-high education. Since education was independently associated with better sexual function, this imbalance may act as a confounder. Nevertheless, the observed difference in healing scores between groups and the positive correlation between healing and sexual function suggest that the lactic acid intervention had an additional beneficial effect beyond demographic factors.

Understanding these challenges is essential in providing support and appropriate guidance to new mothers navigating this sensitive phase.

The statistically significant correlation observed between wound healing quality and postpartum sexual function supports the hypothesis that optimal tissue recovery may facilitate better physical comfort and psychological readiness for resuming sexual activity. However, causality cannot be inferred, and further studies with repeated measures are needed to confirm this relationship.

Future research should explore larger sample sizes, additional clinical parameters (such as pain levels, infection rates, and patient-reported outcomes), and different formulations of lactic acid to determine its precise role in postpartum wound care.

The study is limited by its relatively small sample size. Another limitation of the study is the use of a single follow-up point at 40 days postpartum. This time frame was chosen based on standard local postpartum care protocols, which schedule evaluations around six weeks after delivery. While this timing coincides with the resumption of sexual activity in many women, additional follow-ups at later stages (e.g., 3 or 6 months) would have allowed for a better understanding of long-term outcomes. Future studies should include multiple follow-up points to assess the durability of both wound healing and sexual function recovery. Future research should include larger cohorts and assess long-term outcomes to provide more conclusive evidence on the role of lactic acid in postpartum recovery.

Another key limitation of this study is the absence of randomization and participant blinding. The use of sequential allocation may have introduced selection bias, potentially influencing group comparability despite similar baseline demographics. Additionally, lack of blinding could contribute to evaluation bias, particularly in the subjective reporting of sexual quality of life outcomes. While efforts were made to standardize care and ensure objective data analysis through blinded statistical interpretation, future randomized and blinded trials are needed to validate these findings and minimize methodological bias.

## Conclusions

Lactic acid application was associated with significantly improved episiotomy healing compared to spontaneous recovery. This improvement correlated with better postpartum sexual function. Additionally, education level was identified as an important factor in postpartum sexual quality, while age and number of births were not significant predictors.

While these findings suggest a beneficial role of lactic acid in early postpartum recovery, they are preliminary. Future studies with larger cohorts, multiple follow-up points, and extended observation periods are needed to confirm these results and explore potential long-term benefits.

## Appendices

**Table 6:** Romanian Questionnaire

1. Cât de frecvent simțiți nevoia de a întreține contact sexual? (Poate include dorința de a avea contact sexual, planificarea mentală a unui act sexual, frustrarea de a nu întreține contact sexual):				
Întotdeauna	De Obicei	Uneori	Rar	Niciodată
2. Cât de frecvent ajungeți la orgasm după contactul sexual cu partenerul dumneavoastră?				
Întotdeauna	De Obicei	Uneori	Rar	Niciodată
3. Vă simțiți excitată în timpul contactului sexual cu partenerul dumneavoastră?				
Întotdeauna	De Obicei	Uneori	Rar	Niciodată
4. Cât de satisfăcută vă declarați de varietatea actelor sexuale întreținute?				
Întotdeauna	De Obicei	Uneori	Rar	Niciodată
5. Resimțiți durere în timpul contactului sexual cu partenerul dumneavoastră?				
Întotdeauna	De Obicei	Uneori	Rar	Niciodată
6. Resimțiți senzație de uscăciune în timpul contactului sexual cu partenerul dumneavoastră?				
Întotdeauna	De Obicei	Uneori	Rar	Niciodată
7. Senzația de uscăciune din timpul contactului sexual cu partenerul vă determină să aveți contact sexual mai rar, comparativ cu contactele sexuale înainte de naștere?				
Întotdeauna	De Obicei	Uneori	Rar	Niciodată
8. Încercați să evitați contactul sexual, comparativ cu contactele sexuale avute înainte de naștere?				
Întotdeauna	De Obicei	Uneori	Rar	Niciodată
9. În timpul contactului sexual, experimentați emoții negative, cum ar fi dezgust, frică, vină sau rușine, mai mult decât înainte de a naște?				
Întotdeauna	De Obicei	Uneori	Rar	Niciodată
10. În timpul contactului sexual, partenerul are probleme cu menținerea erecției, comparativ cu contactele sexuale avute înainte de naștere?				
Întotdeauna	De Obicei	Uneori	Rar	Niciodată
11. În timpul contactului sexual, partenerul are probleme cu ejacularea prematură, comparativ cu contactele sexuale avute înainte de naștere?				
Întotdeauna	De Obicei	Uneori	Rar	Niciodată
12. Orgasmele obținute sunt mai intense, comparativ cu contactele sexuale avute înainte de naștere?				
Întotdeauna	De Obicei	Uneori	Rar	Niciodată

**Table 7:** English Translated Questionnaire

1. How often do you feel the urge to have sex? (May include desire for intercourse, mental planning for intercourse, frustration at not having intercourse):				
Always	Usually	Sometimes	Rarely	Never
2. How often you reach orgasm after sexual contact with your partner?				
Always	Usually	Sometimes	Rarely	Never
3. Do you feel aroused during sexual intercourse with your partner?				
Always	Usually	Sometimes	Rarely	Never
4. How satisfied are you with the sexual acts performed?				
Always	Usually	Sometimes	Rarely	Never
5. Do you experience pain during sexual intercourse with your partner?				
Always	Usually	Sometimes	Rarely	Never
6. Do you feel dryness during sexual intercourse with your partner?				
Always	Usually	Sometimes	Rarely	Never
7. Does the feeling of dryness during sexual intercourse with your partner cause you to have intercourse less frequently compared to intercourse before birth?				
Always	Usually	Sometimes	Rarely	Never
8. Do you try to avoid sexual intercourse compared to the sexual contacts you had before the birth?				
Always	Usually	Sometimes	Rarely	Never
9. During intercourse, do you experience negative emotions, such as disgust, fear, guilt, or shame, more than before you gave birth?				
Always	Usually	Sometimes	Rarely	Never
10. During intercourse, does the partner have problems maintaining an erection, compared to intercourse before birth?				
Always	Usually	Sometimes	Rarely	Never
11. During intercourse, does the partner have problems with premature ejaculation, compared to intercourse before birth?				
Always	Usually	Sometimes	Rarely	Never
12. Are the orgasms obtained more intense, compared to sexual contacts before birth?				
Always	Usually	Sometimes	Rarely	Never
